# Pedaling Through Pain: A Case Report of Iliac Artery Endofibrosis in a Competitive Cyclist

**DOI:** 10.7759/cureus.69477

**Published:** 2024-09-15

**Authors:** Adarsh Mallepally, Christopher W Bailey

**Affiliations:** 1 Interventional Radiology, Virginia Commonwealth University School of Medicine, Richmond, USA

**Keywords:** competitive cycling, endofibrosectomy, endurance training athletes' endofibrosis, hip flexion, iliac artery endofibrosis, initimal thickening, lower-extremity claudication, peripheral vascular surgery

## Abstract

Iliac artery endofibrosis (IAE) is a rare cause of leg pain in young, healthy endurance athletes, particularly in male competitive cyclists. The prevailing hypothesis suggests that it is due to mechanical trauma of the iliac artery from long-standing hip flexion. In this case, a 40-year-old male endurance cyclist presented with bilateral thigh pain and worsening leg fatigue upon maximal exertion. Yet, the physical exam at rest was negative for claudication and peripheral pulses were intact. For this reason, IAE is poorly recognized and remains a difficult diagnosis as it requires testing at near-maximal exertion to reveal symptoms. After the patient was referred to the vascular clinic, imaging revealed intimal thickening of the left common iliac artery but with no evidence of stenoses bilaterally. Management typically begins conservatively but true resolution of symptoms can necessitate vascular intervention.

## Introduction

Vascular disease can often be overlooked on the differential for exertional thigh pain in a young, healthy endurance athlete. Iliac artery endofibrosis (IAE) is a rare and poorly recognized condition that is characterized by intimal thickening of the iliac artery in the absence of atheromatous plaque deposits or calcification [1-4]. Cases of this uncommon vascular disease in cyclists were first described decades ago, dating back to 1986 [2]. Although uncommon, IAE is thought to account for 20% of overuse leg injuries in the professional cyclist population [2]. The exact mechanisms are not well established, but it is believed to result from repeated mechanical trauma of the iliac artery [3]. Though IAE can be seen in endurance runners, triathletes, and rugby players, it is most notably associated with cycling because chronic engagement in hip flexion makes them especially susceptible to iliac artery deformation [3,5,6]. In fact, symptoms have been shown to manifest in those who cycle 14,500-20,000 kilometers per year, with an average of 120,000 kilometers completed over their lifetime [1]. The clinical presentation mimics claudication-like symptoms but is reproducible only under intense exertion, making IAE a challenging diagnosis [1,4,5]. In this case study, we aim to provide a comprehensive overview of the current literature surrounding IAE, including its demographics, pathophysiology, clinical presentation, risk factors, diagnostic workup, and management strategies.

## Case presentation

A 40-year-old male competitive cyclist presented with a bilateral “block-like” sensation upon exertion in his lower extremities accompanied by increasing leg fatigue, delimited specifically to the quadriceps and hamstrings. Onset began in the past three months when he began retraining. Despite managing his training load, he started reporting these claudication symptoms as he ramped up his activity. Additionally, he observed a longer duration of soreness and tightness in his thighs with a noticeable loss of power when striving for peak performance, almost mimicking an overtraining session. However, only intense exertion would reproduce symptoms, whereas an easy ride would fail to yield any symptoms. He tried resting for a few days and eventually took over a month off before returning to intense training, but the sensation persisted. He denied any prior issues with repeated maximal intensity efforts while cycling. The physical exam was notable for tenderness of the lower left psoas muscle and mild lumbar scoliosis. He had intact pulses and 5/5 strength throughout. He denied lower extremity paresthesia. He had no significant prior medical or surgical history and denied smoking and substance use. 

**Figure 1:**
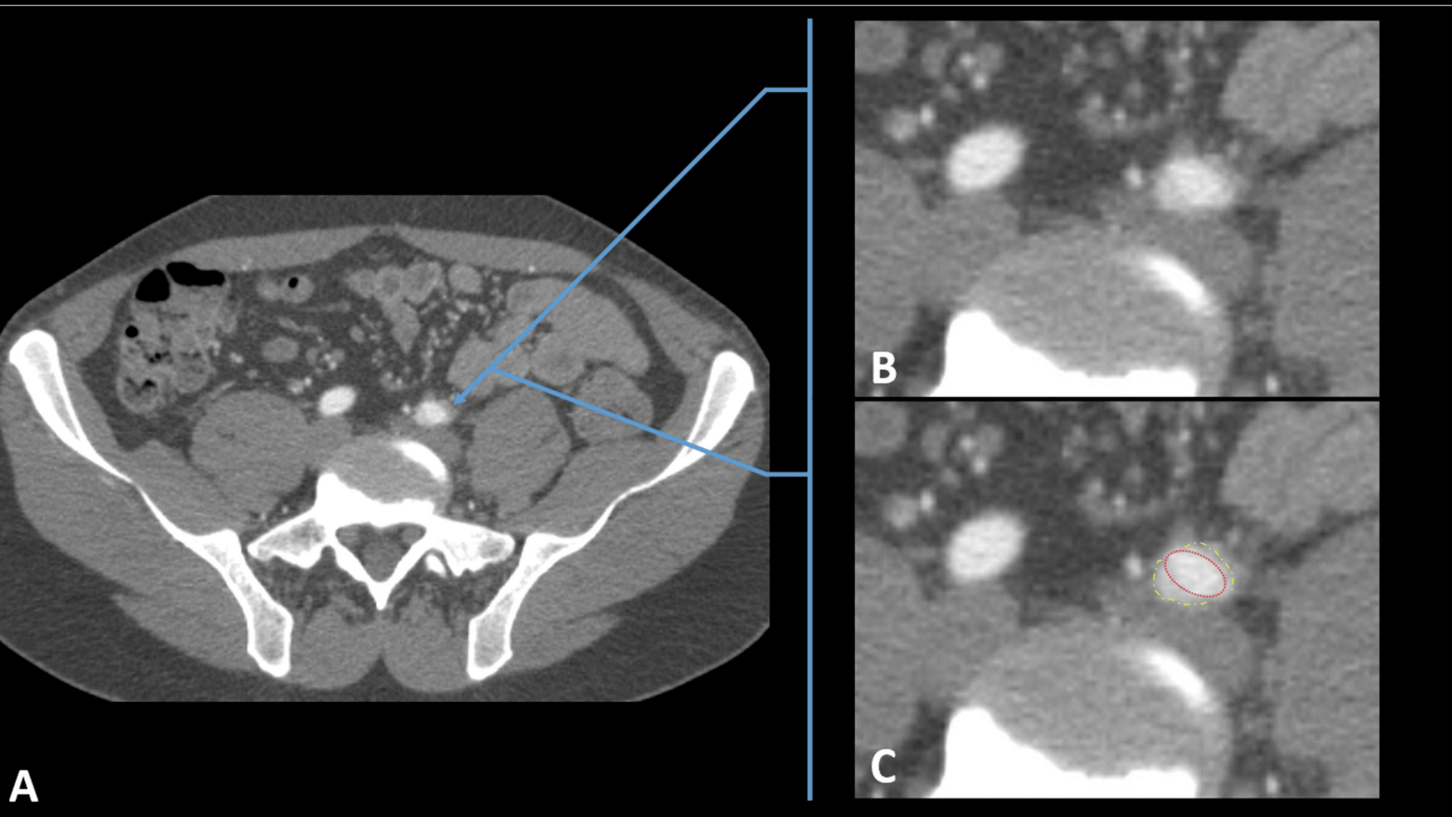
CT angiography of the pelvis in the axial plane Contrast-enhanced CT imaging of the pelvis in the early arterial phase provides evaluation of the arteries. Specifically, in this cross section, the common iliac artery (CIA) is shown bilaterally relative to surrounding pelvic viscera in a non-magnified view (A). (B and C) Cropped magnified axial images created from (A). (C) Annotations of the normal vessel (red perforated oval) versus the abnormal fibrotic change surrounding the vessel (yellow perforated oval).  The CIA on the left is pathologic, whereas the CIA on the right is normal (A-C).

**Figure 2:**
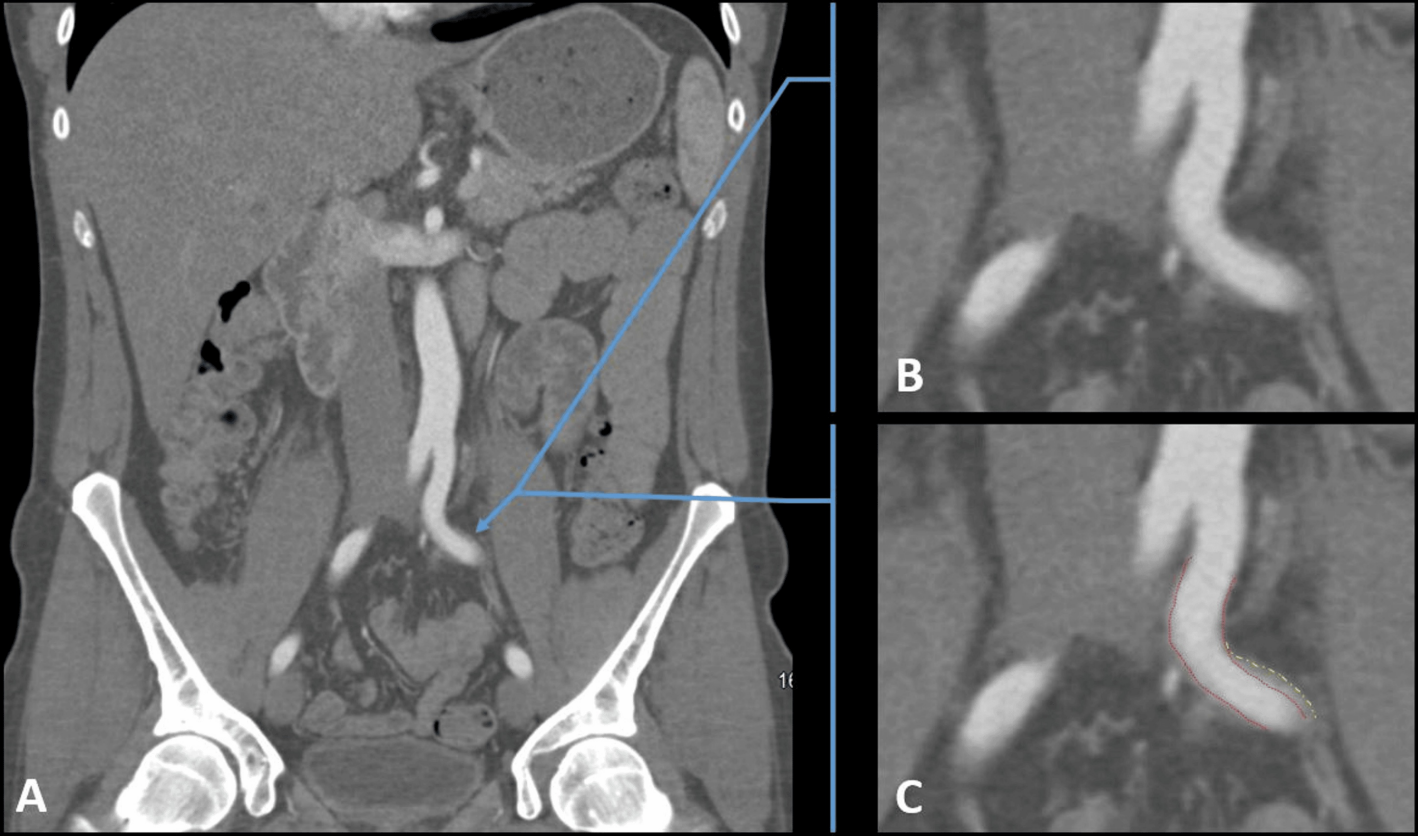
CT angiography of the pelvis in the coronal plane Contrast-enhanced CT imaging of the pelvis in the early arterial phase provides evaluation of the arteries. Specifically, in this cross section, the common iliac artery (CIA) is shown bilaterally relative to surrounding pelvic viscera in a non-magnified view (A). (B and C) Cropped magnified coronal images created from (A). (C) Annotations of the normal vessel (red perforated oval) versus the abnormal fibrotic change surrounding the vessel (yellow perforated oval).  The CIA on the left is pathologic, whereas the CIA on the right is normal (A-C).

The initial workup included a diagnostic pelvic X-ray and an ankle-brachial index (ABI) at rest, both of which came back normal. Physical therapy of the lumbar spine was recommended, but the exertional thigh pain still remained. The patient was subsequently referred to a vascular clinic and scheduled for a pelvic computed tomography angiography (CTA) as seen in Figures 1A-1C, 2A-2C to evaluate for IAE. The CTA demonstrated subtle intimal thickening in the left common iliac artery (CIA) but without plaque, calcification, dissection, or peri-arterial abnormality. Surprisingly, there was also no evidence of hemodynamically significant stenoses in the aorta, iliac, or proximal femoral arteries bilaterally. In order to further evaluate the vascular status of his legs physiologically and anatomically, he was arranged to undergo an exercise ABI study using his own cycling equipment to reproduce the symptoms. Unfortunately, the patient was lost to follow-up at this time.

## Discussion

IAE is a rather uncommon and poorly recognized cause of leg pain in otherwise healthy presenting patients. This condition typically affects young male endurance athletes, particularly competitive cyclists, under the age of 40 [4,5]. In fact, an estimated 10-20% of the competitive cyclist population younger than 40 is thought to be affected by IAE, of which 80% are accounted for by males [1]. IAE’s predilection for males is likely a direct consequence of more men participating in endurance sports such as cycling, running, and triathlons [5,6]. Although the mechanistic underpinnings of IAE are unclear, the current literature may provide insight as to why the competitive cyclist population is at greatest risk.

The pathophysiology of IAE is hypothesized to involve repeated direct mechanical trauma and deformation of the iliac artery due to hip flexion [3]. Hypertrophy of the psoas muscle from overuse is also thought to play a role in IAE, inducing excessive stretch and elongation of the arterial wall [3]. Together, they can lead to pathological thickening of the tunica intima in the iliac artery [1,2,7]. Thus, competitive cycling is believed to be a possible etiology due to its role in the long-term consequences of hip flexion. Due to its inherent position of attachment to the psoas muscle, the external iliac artery (EIA) is most commonly injured and subject to intimal thickening [4]. Eventually, progressive arterial stenosis in the absence of atheromatous plaques can occur, resulting in the claudication symptoms seen in patients afflicted with IAE [1,3,4]. Another key player could involve vasospasm of the iliac artery following intense exercise. Whereas unaffected arteries vasodilate in response to exercise, the intimal thickening renders the vessels unable to effectively mediate blood perfusion and instead vasoconstricts, leading to claudication as well [4,8]. Both mechanisms support the current hypothesis of the pathophysiology behind IAE. If left untreated, there is some evidence suggesting that IAE may increase a patient’s predisposition to atherosclerotic plaques and thrombosis [2,9].

The clinical presentation of IAE is characterized by unilateral and reproducible pain upon maximal exertion localized to the thigh, buttocks, and calf that quickly resolves within minutes of rest [2,5]. Cramping, swelling, and numbness are also common symptoms [4]. Most importantly, physical exam and pulses will be normal at rest because abnormalities are only evidenced upon exertion, making it difficult to diagnose [5]. It also remains a poorly recognized condition because vascular disease is not expected in young athletes and, thus, the pain can be mistakenly attributed to musculoskeletal or neurological etiologies [1]. For these reasons, the diagnosis of IAE often occurs only after failed physical therapy, delaying the time to diagnosis to anywhere from 12 to 41 months [1,5].

There are also some anatomical abnormalities frequently found in elite endurance athletes with IAE that are considered risk factors. For example, having a fixated iliac arterial branch overlying the psoas muscle or surrounding adherent fibrous tissue can lead to a kinking phenomenon, increasing the chance of possible vessel trauma [3,10]. Moreover, an excessively long iliac artery can lead to increased tortuosity during hip flexion, resulting in kinking as well [3,5,11]. Lastly, endurance athletes with IAE are more likely to have a larger thigh circumference on a particular leg [4]. Though the mechanism is unclear, this asymmetry may explain why lower extremity claudication symptoms and fatigue typically occur unilaterally in those with IAE [4].

However, the patient described in this case uniquely demonstrated bilateral symptoms. On top of this, the pelvic CTA showed intimal thickening of the CIA instead of the more prevalent EIA. There was also no evidence of significant stenoses. These uncommon findings illustrate the variety in clinical presentation in those with IAE, adding another layer of complexity to an already rare condition.

Diagnosis of IAE requires a combination of detailed history-taking, thorough physical exam, and testing and imaging modalities [1]. Though physical exam at rest can be futile, sometimes weakened distal pulses can be palpated and iliac artery murmurs during hip flexion and extension can be auscultated [1]. An abnormal ABI showing decreased blood flow in the affected lower extremity after maximal exertion compared to a normal ABI at rest is often one of the easier and inexpensive methods [2,4]. Doppler ultrasound demonstrating a significantly higher peak systolic velocity or a duplex ultrasound revealing endofibrosis and anatomical kinking can similarly be used as a quick tool to evaluate for IAE [4,5]. The most robust form of diagnosis is likely a pelvic CTA or magnetic resonance angiography, which can confirm intimal thickening, stenosis, and any kinking of the iliac artery [4,5]. Although not commonly done, histopathological analysis of the intima would show dense accumulation of loose connective tissue composed of collagen, elastin, fibroblasts, and smooth muscle cells [2,3]. This is in stark contrast to a fatty or calcified atherosclerotic plaque.

A conservative approach to management is typically offered first. Limiting intense physical activity and minimizing hip flexion are of utmost priority [4]. Specifically with respect to cycling, this entails positional adjusting such as raising the handlebars and bringing the seat forward [5]. It is also recommended to avoid pulling the pedal upwards to reduce psoas muscle hypertrophy [5]. Physical therapy tends to be offered at this time as well, but the current literature does not provide any evidence that it relieves symptoms. Should symptoms persist after this conservative approach, vascular surgical intervention appears to be the most effective next step [1,2,4,12]. The gold standard for IAE is an endofibrosectomy with patch angioplasty to remove the fibrosed portion of the iliac arterial wall and prevent narrowing, all while minimizing complications [4,5,7]. It Is important to note that traditional procedures used to treat atherosclerosis such as balloon angioplasty and stents are not indicated and result in higher recurrence rates [7,12,13]. This is because endofibrotic lesions are distinctly elastic and can recoil easily just days after the procedure, leaving the patient susceptible to embolisms [2,3,7]. In cases with anatomical abnormalities such as an excessively long iliac artery, a formal resection with end-to-end anastomosis or interposition great saphenous vein graft is recommended [7]. Or, in cases with kinking from fixated iliac arterial branches, surgical release of tight fibrous tissue may be optimal [7].

## Conclusions

Though IAE is a relatively rare vascular disease, it should be considered when evaluating reproducible thigh pain with claudication symptoms upon exertion in young, healthy endurance athletes. Typically seen in cyclists, IAE is a direct result of the anatomical location of the EIA overlying the psoas muscle, leading to intimal thickening. Excessively long or fixated iliac arterial branches may be anatomical risk factors for IAE. Though unilateral claudication is common, this case study illuminates key differences in the classic presentation, such as bilateral pain and thickening of the CIA. Most importantly, this report serves to emphasize that IAE is often poorly recognized and difficult to diagnose, which stems from a multitude of reasons. These include a physical exam negative for claudication at rest with intact peripheral pulses, symptoms reproducible only upon maximal exertion, the inherent rarity of a vascular condition in young patients, subtle variances in clinical presentation, and non-specific histories relayed by patients. If IAE is suspected, a pelvic CTA demonstrating intimal thickening is needed to confirm the diagnosis. Currently, endofibrosectomy with patch angioplasty is the indicated treatment for IAE with persistent symptoms.
